# Proteomics analysis of human tears from aqueous-deficient and evaporative dry eye patients

**DOI:** 10.1038/srep29629

**Published:** 2016-07-20

**Authors:** Natarajan Perumal, Sebastian Funke, Norbert Pfeiffer, Franz H. Grus

**Affiliations:** 1 Department of Ophthalmology, University Medical Center of the Johannes Gutenberg University Mainz, Mainz, Germany

## Abstract

Despite the high global prevalence of dry eye syndrome (DES), the fundamental processes underlying this pathology remain largely unexplored. Therefore, this study endeavoured to investigate in-depth the tear proteome of DES patients employing the mass spectrometry (MS)-based proteomic strategies. Eighty patients were recruited and subdivided into three major DES subgroups, which are the aqueous-deficient (DRYaq), evaporative (DRYlip) and a combination of the two (DRYaqlip), as well as healthy subjects (CTRL). Discovery proteomics strategy was employed to identify large number of significantly differentially expressed tear proteins in DRYlip *vs*. CTRL, DRYaq *vs*. CTRL and DRYaqlip *vs*. CTRL with 22, 58 and 67 proteins, respectively. Biological functional analysis demonstrated for the first time that various metabolic processes were highly expressed in DRYaq and DRYaqlip, which might modulate various other known processes, especially the inflammatory and immune processes. Targeted proteomics strategy verified that 13 major proteins were differentially expressed in specific DES subgroups, comprising of PRR4, ZG16B, SCGB2A1, DMBT1, PROL1, LACRT, ALDH3A1, ENO1, TF, S100A8, S100A9, PEBP1 and ORM1. In conclusion, this study had explored in-depth the pathology of DES by unravelling various new fundamental processes and the major proteins responsible for the maintenance of tear film stability.

It is well- recognized that the dry eye syndrome (DES) is a common yet deplorable pathology of the ocular surface and tears that arises from a plethora of factors, which could result in decreased visual acuity, ocular discomfort and tear film instability with potential damage risk to the cornea^1^. The study of DES has become a subject of much interest due to the high rate of occurrence in different ethnic groups, gender (preponderance in women), age (>50 years old) and other ocular surface-affecting systemic diseases (especially Sjögren’s syndrome, diabetes mellitus, pterygium and allergy)^2^,^3^,^4^,^5^,^6^,^7^. In recent years, assessment studies in several Western and Asian countries reported a high global prevalence of this pathology, ranging from 2% to over 50% of the world population^8^,^9^,^10^,^11^,^12^,^13^,^14^,^15^,^16^,^17^. Apart from afflicting one out of every two elderly in some populations, this condition also poses a substantial economic burden on the health system^18^,^19^. There are various underlying aetiologies for DES and the major causes of DES are a consequence of aqueous tear-deficiency (DRYaq) or due to changes in the lipid phase (DRYlip) of the tear film^1^.

To date, many studies have identified the differentially expressed proteins in tears of DES patients. Grus *et al*. had profiled tear proteins from patients with DES and they found a significant increase in S100-A8 protein (S100A8) and decrement of proline-rich protein 4 (PRR4), lysozyme C (LYZ), proline-rich protein 3 and α-1-antitrypsin^20^. The investigation of the tear proteome of patients with DRYaq, DRYlip and a combination of the two (DRYaqlip) compared to healthy controls (CTRL) by Boehm *et al*. revealed that specific alterations of the tear proteome reflect the different clinical phenotypes of DES^21^. They also demonstrated that PRR4 expression level was decreased and, 3 other proteins, including S100A8, increased significantly in both DRYaq and DRYaqlip groups. However, the tear proteome of DRYlip patients strongly deviated from the DRYaq or DRYaqlip groups and demonstrated only slight alterations. Meanwhile, Soria *et al*. showed that 6 proteins were found to be increased and 9 proteins to be decreased in abundance in both DRYaq and DRYlip^22^. Zhou *et al*. employed the isobaric Tags for Relative and Absolute Quantitation (iTRAQ) quantitative proteomics strategy to identify potential tear biomarkers for DES and they found an increase of 6 proteins and a decrease of 4 proteins^23^. Similarly, utilizing the iTRAQ tool, Srinivasan *et al*. investigated the differentially expressed tear proteins in patients with mildly symptomatic aqueous deficiency (MDE), symptomatic aqueous deficiency (MSDE) and a combination group (MXDE)^24^. In addition to the decreased abundance of commonly reported proteins, such as LYZ, lipocalin-1 (LCN1) and prolactin-inducible protein (PIP) across all DES sub-groups, their finding demonstrated a number of proteins that were significantly differentially expressed in subgroups of DES. Other DES studies have investigated Sjögren’s syndrome (SS) dry eye, which is a systemic autoimmune disease characterized by compromised lacrimal and salivary gland functions that causes severe dry eye and dry mouth^6^,^25^.

The aforementioned DES studies only reported limited number of differentially expressed proteins for the pathology, largely due to the limitations in the methodology employed. These studies had employed various mass spectrometry (MS)-based proteomic strategies, which have become a progressively powerful technology for the identification and quantifications of tear proteins in different ocular pathologies^5^,^20^,^21^,^23^,^26^,^27^,^28^,^29^,^30^,^31^,^32^,^33^,^34^,^35^. Recently, protein quantification with isotopic labels (e.g. iTRAQ) has been commonly utilized for tear protein studies due to their quantitative accuracy, coverage and robustness^23^,^24^. However, despite their usefulness, they inherently require extra preparation steps that consequently increase the complexity and often prohibit their use for biological questions that require the detection of subtle yet vital proteome changes, mostly the low abundant tear proteins. On the contrary, label-free quantification (LFQ) is the simplest and most economical strategy that can be utilized for in-depth analysis of the differentially expressed proteins in large sample size with high precision^36^. Additionally, the development and improvement of the existing algorithms and software tools, especially, MaxQuant software suite, have provided an effective solution for LFQ analysis, one that achieves state-of-the-art quantification accuracy and coverage^37^,^38^.

Therefore, in-depth investigation of the differentially expressed proteins in tear proteome of DES patients will potentially contribute to better understanding of the fundamental processes of this pathology. Hence, the main objective of the present study was to identify the differentially expressed proteins in the major DES subgroups, which are responsible for the maintenance of tear film stability. The tear proteome profiles of DES subgroups were analysed employing the bottom-up label-free MS-based proteomics strategies. The ultimate outcome of this study is envisaged to unravel the intricate regulation profiles of specific proteins responsible for the maintenance of tear film stability and pin-point specific alteration(s) in the different DES subgroups.

## Results

### Identification of the differentially expressed tear proteins from DES patients

**Figure 1:**
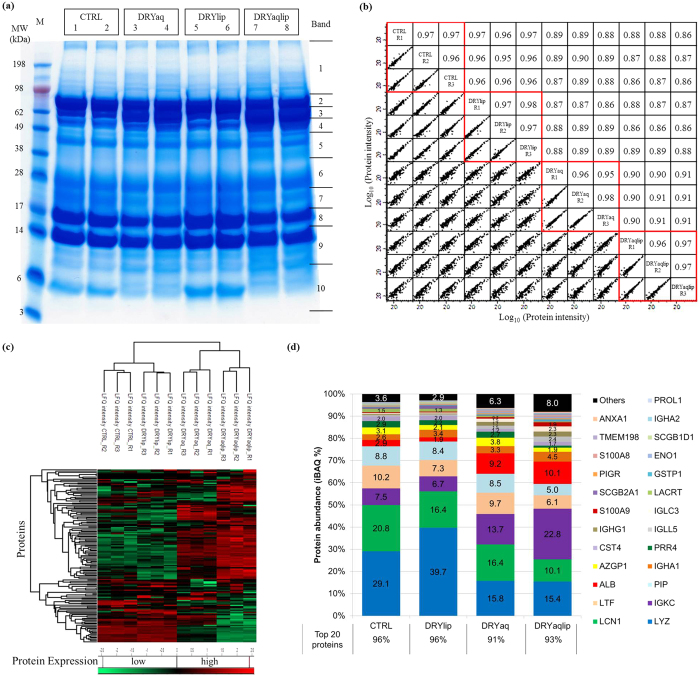
Discovery proteomics analysis *via*1DE & LC-ESI-MS/MS strategy reveals the characteristics of the tear proteome of DES patients. (**a**) Representative tear protein profiles of the DES subgroups and CTRL resolved in 1DE gel after colloidal blue staining. (**b**) The degree of variances in the proteome between the DES subgroups and CTRL investigated by Pearson correlation analysis. (**c**) The heat map shows hierarchical clustering analysis of the 200 tear proteins that separates the designated groups into two main clusters. (**d**) The bar chart shows the degree of protein abundance in DES subgroups and CTRL.

The representative tear protein profiles of DES subgroups and CTRL resolved in 1DE gel are illustrated in Fig. 1a. Notable differences were observed at band three, four, seven and ten, which represent serum albumin (ALB), Ig alpha-1 chain C (IGHA1), PRR4 and mammaglobin-B (SCGB2A1), respectively. A total of 200 proteins were detected by the discovery approach as presented in Supplementary data 1A. In order to reveal the differentially expressed proteins in the DES subgroups compared to CTRL, LFQ values of the identified proteins extracted from MaxQuant analysis were used for statistical analysis utilizing the Perseus software. Basically, there is good technical reproducibility of the entire experimental workflow, which reveals Pearson correlation of 0.96 ± 0.01 for DRYaq and 0.97 ± 0.01 for CTRL, DRYlip and DRYaqlip as shown in Fig. 1b. On average, correlation between DRYlip *vs*. CTRL was 0.96 ± 0.01, and slightly lower correlations were observed between the DRYaq *vs*. CTRL (0.89 ± 0.01) and DRYaqlip *vs*. CTRL (0.87 ± 0.01) groups. Hence, this demonstrated high similarities between the proteome of CTRL and DRYlip, as well as between DRYaq and DRYaqlip. In addition, this analysis indicates that reproducible data were generated from the pooled tear samples, which enabled further statistical analysis. A heat map with unsupervised hierarchical clustering of the data was generated and resulted in two major clusters, which are cluster 1 comprising CTRL and DRYlip, and cluster 2 comprising DRYaq and DRYaqlip, as shown in Fig. 1c. In order to identify a subset of proteins that significantly differentiate the DES subgroups compared to CTRL, a two samples t-test (P < 0.01) was performed. The summary of the significantly differentially expressed proteins in the different DES subgroups compared to CTRL is as tabulated in Supplementary data 1B. The total number of proteins that were significantly differentially expressed in the DRYlip *vs*. CTRL was 22 proteins, 58 proteins in the DRYaq *vs*. CTRL and 67 proteins in the DRYaqlip *vs*. CTRL. Next, intensity-based absolute quantification (iBAQ) values of the identified proteins extracted from MaxQuant analysis were used to elucidate the abundance of the identified proteins between DES subgroups and CTRL. Figure 1d shows that only top 20 abundant proteins from the total of 200 proteins identified make up almost 95.52 ± 0.32%, 96.36 ± 0.65%, 91.0 ± 0.13% and 92.55 ± 0.56% of the total abundance in CTRL, DRYlip, DRYaq and DRYaqlip, respectively (complete data in Supplementary data 1A). Moreover, LYZ, LCN1, lactotransferrin (LTF) and PIP make up as much as 60% of the total abundance of the CTRL tears. Generally, the aforementioned iBAQ analysis revealed that most of the significantly differentially expressed proteins were classified as low abundant proteins (lower than 0.5%) in this study, as shown in Supplementary data 1B.

### Functional classification and protein interaction network analysis of the significantly altered proteins

**Figure 2:**
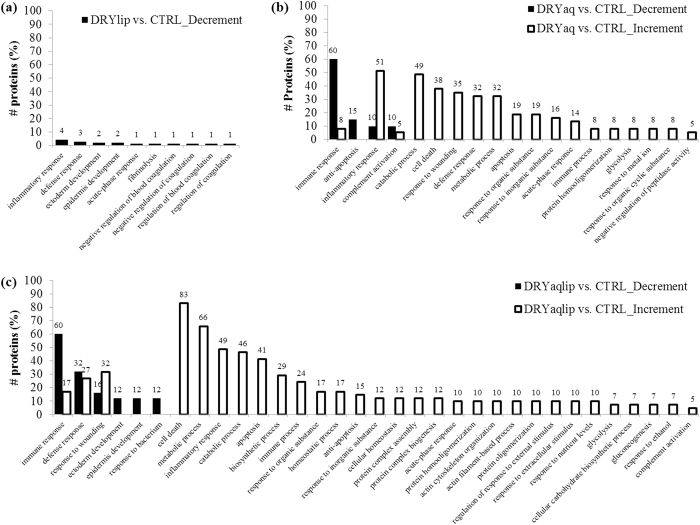
The bar charts shows the over-represented GOBP terms associated with the differentially expressed proteins analysed employing the DAVID tool in (**a**) DRYlip *vs.* CTRL, (**b**) DRYaq *vs.* CTRL and (**c**) DRYaqlip *vs.* CTRL.

**Figure 3:**
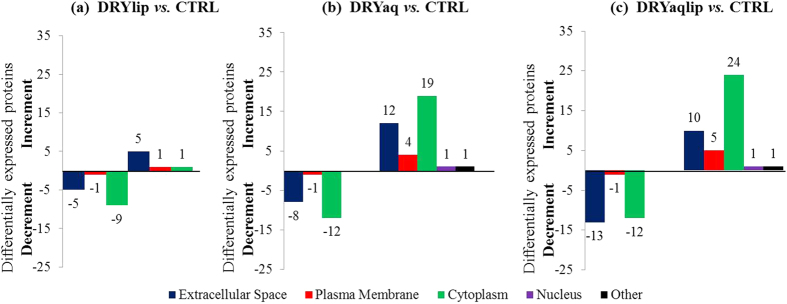
The bar charts shows the over-represented GOCC terms associated with the differentially expressed proteins analysed employing the Ingenuity Pathways Analysis software in (**a**) DRYlip *vs.* CTRL, (**b**) DRYaq *vs.* CTRL and (**c**) DRYaqlip *vs.* CTRL.

**Figure 4:**
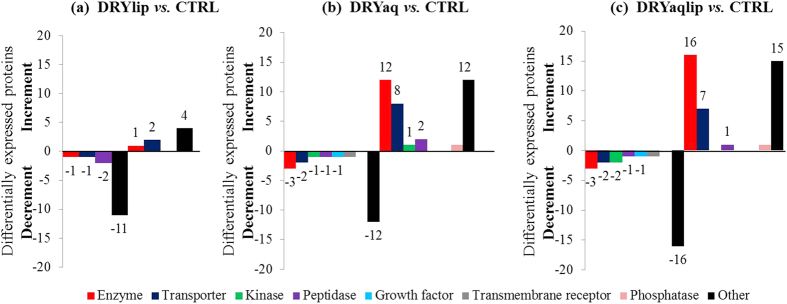
The bar charts shows the over-represented molecular type terms associated with the differentially expressed proteins analysed employing the Ingenuity Pathways Analysis software in (**a**) DRYlip *vs*. CTRL, (**b**) DRYaq *vs*. CTRL and (**c**) DRYaqlip *vs*. CTRL.

**Figure 5:**
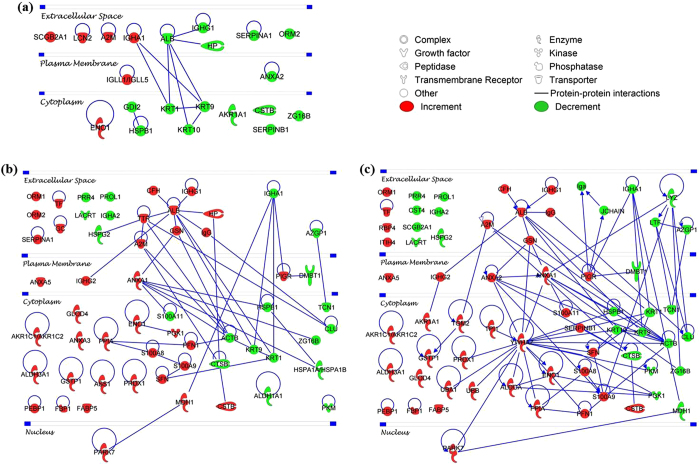
Networks of PPI of the significantly differentially expressed tear proteins analysed employing the Ingenuity Pathways Analysis software in (**a**) DRYlip *vs*. CTRL, (**b**) DRYaq *vs*. CTRL and (**c**) DRYaqlip *vs*. CTRL.

To determine which biological processes were most affected in specific DES subgroups, the over-represented gene ontology biological process (GOBP) terms associated with the differentially expressed proteins were analysed employing the Database for Annotation, Visualization and Integrated Discovery (DAVID) tool. Several major biological process categories were observed to be decreased in DRYlip *vs*. CTRL, mainly inflammatory response (4%), however they were identified with lower percentage, as shown in Fig. 2a. On the contrary, 4 major biological processes categories were observed to be decreased in DRYaq *vs*. CTRL, especially immune responses (60%), as shown in Fig. 2b. Consequently, 18 major biological processes were found to be increased in this group and large percentage of them are involved in inflammatory response (51%), catabolic process (49%), cell death (38%), response to wounding (35%), defence response (32%), metabolic process (32%) and apoptosis (19%). Six major biological processes were found to be decreased in DRYaqlip *vs*. CTRL, especially immune response (60%) and defence response (32%), as shown in Fig. 2c. Meanwhile, as many as 30 biological processes were found to be increased in this group and most highly ranked were involved in cell death (83%), metabolic process (66%), inflammatory response (49%), catabolic process (46%) and apoptosis (41%). The complete list of the over-represented GOBP terms of the differentially expressed proteins in DES subgroups compared to CTRL can be found in Supplementary data 1C. The characteristic of the differentially expressed proteins were further analysed, the over-represented gene ontology cellular component (GOCC) terms, molecule types and protein-protein interaction (PPI) networks were generated using Ingenuity Pathways Analysis. The over-represented GOCC terms of the significantly decreased proteins in DRYlip *vs*. CTRL were 5 extracellular space proteins and 9 cytoplasm proteins as well as increased in other 5 extracellular space proteins as shown in Fig. 3a. Meanwhile, the most significantly decreased proteins in DRYaq *vs*. CTRL were 8 extracellular space proteins and 12 cytoplasm proteins as well as drastic increased in other 12 extracellular space proteins and as many as 19 cytoplasm proteins as shown in Fig. 3b. Almost similar profiles were observed in DRYaqlip *vs*. CTRL, decreased of 13 extracellular space proteins and 12 cytoplasm proteins as well as increased in other 10 extracellular space proteins and 24 cytoplasm proteins were identified as shown in Fig. 3c. The molecular characteristics analysis of the differentially expressed proteins showed that many of these proteins are enzyme (23 proteins), transporter (12 proteins), kinase (2 proteins) peptidase (3 proteins), growth factor (1 protein), transmembrane receptor (1 protein), phosphatase (1 protein) and other (36 proteins), as shown in Supplementary data 1B. Generally, only numerous proteins of enzyme, transporter and peptidase were observed to be differentially expressed in DRYlip *vs*. CTRL, as shown in Fig. 4a. On the contrary, large preponderances of enzyme (12 proteins) and transporter (8 proteins) proteins were observed to be increased in DRYaq *vs*. CTRL, as shown in Fig. 4b. Similarly, as many as 16 enzymes and 7 transporter proteins were observed to be increased in DRYaqlip *vs*. CTRL, as shown in Fig. 4c. Besides, many of the differentially expressed proteins in all the DES subgroups were annotated as other, many of them of unknown characteristics, namely, PRR4 and zymogen granule protein 16 homolog B (ZG16B). The summary of the PPI networks of the differentially expressed proteins according to their GOCC terms and molecule types characteristics are as shown in Fig. 5 (the complete lists of PPI networks for each comparison are reported in Supplementary data 1D). Figure 5a demonstrated that there are only 20 direct PPI networks between cytoplasm and extracellular space proteins in DRYlip *vs*. CTRL. On the contrary, as many as 74 and 169 PPI were observed in DRYaq *vs*. CTRL and DRYaqlip *vs*. CTRL, as shown in Fig. 5b,c, respectively. In DRYaq *vs*. CTRL, the proteins with the highest number of direct PPI networks were transthyretin (TTR) (6), S100A8 (3), cathepsin B (CTSB) (3), gelsolin (GSN) (3), polymeric immunoglobulin receptor (PIGR) (3) and 14-3-3 protein sigma (SFN) (3). In DRYaqlip *vs*. CTRL, the proteins with the highest number of direct PPI were 14-3-3 protein zeta/delta (YWHAZ) (23), protein S100-A9 (S100A9) (9), ALB (8), annexin A1 (ANXA1) (7), LYZ (6) and PIGR (6). Generally, the most significantly altered PPI networks and their related top diseases and functions for the DRYlip *vs*. CTRL, DRYaq *vs*. CTRL and DRYaqlip *vs*. CTRL were “dermatological diseases and conditions, developmental disorders”, “cellular movement, immune cell trafficking, metabolic disease” and “antimicrobial response, inflammatory response, humoral immune response”, respectively, as tabulated in Table 1.

### Verification of the major differentially expressed tear proteins from DES patients

Finally, a rapid and robust approach *via* in-solution digestion of the pooled tear samples and accurate inclusion mass screening (AIMS) analysis was utilized to verify the major differentially expressed tear proteins in DES subgroups compared to the CTRL. Table 2 shows the detailed AIMS analysis of the signature peptides based on representative precursor ions for specific proteins (complete data in Supplementary data 1E). This analysis ascertained the differentially expressed profiles of 13 proteins in the DES subgroups compared to CTRL. Among the 13 differentially expressed proteins identified, PRR4, ZG16B and proline-rich protein 1 (PROL1) were found to be significantly decreased in both DRYaq and DRYaqlip subgroups but only slightly decreased in abundance in the DRYlip subgroup. SCGB2A1 and deleted in malignant brain tumors 1 protein (DMBT1) were found to be significantly decreased in both DRYaq and DRYaqlip subgroups. Extracellular glycoprotein lacritin (LACRT) was found significantly decreased only in DRYaqlip subgroup. On the contrary, S100A8, S100A9 were found to be significantly increased in both DRYaq and DRYaqlip subgroups but only slightly increased in abundance in the DRYlip subgroup. Alpha-enolase (ENO1), serotransferrin (TF), phosphatidylethanolamine-binding protein 1 (PEBP1) and alpha-1-acid glycoprotein 1 (ORM1) were found to be significantly increased in both DRYaq and DRYaqlip subgroups. Aldehyde dehydrogenase, dimeric NADP-preferring (ALDH3A1) was found significantly increased only in DRYaqlip subgroup. Two signature peptides for PRR4 were utilized in the AIMS analysis because these two peptides are essential for exact identification and quantification of this protein^35^.

## Discussion

This study had unravelled as many as 79 differentially expressed tear proteins in the DES subgroups compared to CTRL based on the LFQ analysis. Similar expression profiles of the 37 of proteins from this list were already demonstrated in other studies associated with dry eye with/without systemic diseases, as tabulated in Supplementary data 1F, thereby, corroborating with the present results. This study also identified 42 novel proteins associated with DES, and this supports the relevance of the quantitative tear proteomics to identify novel differentially expressed proteins for specific DES.The functional classification and protein interaction network analysis of the significantly altered proteins suggest that the development of DES is a complicated process involving proteins of multiple biological functions. In this study, although 22 tear proteins were differentially expressed in DRYlip *vs*. CTRL, they were only differentially expressed in a lesser degree and most of them are involved in the dermatological diseases and conditions. On the contrary, the differentially expressed proteins in DRYaq *vs*. CTRL and DRYaqlip *vs*. CTRL demonstrated the involvement of mostly extracellular space and cytoplasm proteins, which consisted largely of enzymes and transporter protein types. Basically, the PPI networks analysis demonstrated the involvement of various biological processes and diseases, especially the cellular movement, immune cell trafficking, antimicrobial response, inflammatory response, humoral immune response, metabolic disease and neurological disease. Based on the outcomes of this study, it could be postulated that the pathological mechanisms underlying DRYaq and DRYaqlip are driven by tear film instability caused largely by the decrement of specific extracellular space and cytoplasm proteins, which mainly induces the activation of inflammatory, immune defence, cell death and apoptosis mechanisms. These host-defence mechanisms act in concert to maintain homeostasis of the tear film following the disease insult^1^. These diverse functions are bio-energetically expensive and require precise control of cellular metabolic pathways^39^. Collectively, these results reveal for the first time that high numbers of metabolic enzymes are expressed, preferentially to fuel the cell fate decisions and effector functions of the aforementioned metabolic processes.

In this study, for the first time, the iBAQ analysis demonstrated that the top 20 abundant proteins are accountable for approximately 90% of the total abundance of the tear proteome. This analysis also revealed that most of the significantly differentially expressed proteins were classified as low abundant proteins. For that reason, only 13 major proteins were successfully verified employing the AIMS strategy in DES subgroups compared to CTRL, which composed of PRR4, ZG16B, SCGB2A1, DMBT1, PROL1, LACRT, ALDH3A1, ENO1, TF, S100A8, S100A9, PEBP1 and ORM1. Drastic decrement in abundance of truncated PRR4 in the DRYaq subgroup has been demonstrated previously^6^,^21^,^24^,^26^. In addition, Boehm *et al*. and Nichols *et al*. have demonstrated similar decrement profile of PRR4 in the DRYaqlip subgroup and in contact lens users associated with DES (DRY_CL), respectively^20^,^21^. Decreased PRR4 expression level was also widely documented in recent studies of DES associated with systemic diseases, namely SS, Stevens-Johnson syndrome (SJS) and rheumatoid arthritis (RA)^6^,^7^,^25^. It is noteworthy that similar decrement patterns of PRR4 were documented in the tears of patients with other systemic diseases such as in diabetic proliferative retinopathy, thyroid-associated orbitopathy and multiple sclerosis^40^,^41^,^42^. Interestingly, although the decreased expression profiles of PRR4 is well documented to be associated with DES and the attribution to the functional relevance of the lacrimal gland, the exact biological functions of this protein in tears remains to be characterized. In addition, our recent study had characterized PRR4 as a complex protein with multiple isoforms and post-translational modifications, which provides an fundamental information for determination of the functional consequences in the disease state^35^. On the other hand, the decrement of ZG16B was only demonstrated by Srinivasan *et al*. in tears of MSDE patients^24^. In addition, Salvisberg *et al*. reported decrement of ZG16B in tears of patients with multiple sclerosis^42^. However, both studies did not verify the ZG16B expression profiles employing orthogonal methods, mainly owing to the unavailability of specific functional antibodies. The precise biological function of ZG16B is also largely unknown in tears. Up till now, ZG16B is known as a secretory lectin protein that is proposed to play a regulatory role in intestinal goblet cells and in the pancreatic acinar cells to stimulate “flushing out” of the granule content during exocytosis^43^,^44^. Furthermore, this secreted protein was also assumed to play an important role in the maintenance of the inflammatory state in cancer tissue^45^,^46^,^47^,^48^,^49^. It is important to highlight here that similar to the findings of this study, both ZG16B and PRR4 were found to be distinctly decreased in the tear samples of patients with multiple sclerosis, a progressive neurological disability processes^42^. On the contrary, in our recent study, both PRR4 and ZG16B were found to be significantly increased in abundance in reflex tears, a stimulus by neurological processes^50^. Taken together, the decrement of PRR4 and ZG16B in tears of DES patients could be hypothesized to be associated with the impairment of the neurological processes in the lacrimal gland. Next, similar to the findings of the present study, Srinivasan *et al*. also documented decrement of PROL1 in the MSDE^24^. The exact function of PROL1 is also still largely unknown. Recently, Dufour *et al*. reported that the immunoreactive opiorphin (QRFSR-peptide), a mature secretory peptide product of the PROL1 gene, is secreted primarily by lacrimal gland tears at the highest physiological rates (~200 ng/ml) in healthy volunteers^51^. It has been suggested that the potential role of opiorphin is in modulating lacrimal fluid homeostasis by increasing enkephalin bioavailability in case of certain causes of epiphora. Opiorphin has also been proposed to play paracrine and/or autocrine roles in the lacrimal system and at the ocular surface. Similar decrement in abundance of SCGB2A1 in DRYaq group was also confirmed by Soria *et al*. and Srinivasan *et al*.^22^,^24^. Although the exact physiological function of SCGB2A1 is still not known, it is hypothesized to play a crucial role as an anti-inflammatory agent owing to its association as a member of the uteroglobin family^52^. Next, DMBT1 was found for the first time to be significantly decreased in abundance in DRYaq and DRYaqlip subgroups in this study. It is well documented that this protein plays critical roles in the epithelial differentiation, cellular immune defence and mucosal defence system^53^,^54^,^55^. Decrement abundance of LACRT was also documented in various studies associated with DES, namely in the DRYaq, SS, SJS, RA, MDE, MSDE and DRY_CL^6^,^20^,^24^,^25^. Recent studies demonstrated that this secretory glycoprotein might play an imperative function in secretion and renewal of ocular surface and lacrimal epithelia^56^,^57^. In this study, protein S100A8 and S100A9 were found to be increased in abundance in all the DES subgroups, and they discriminated best the DRYaq and DRYaqlip from the CTRL. Decrement in abundance of S100A8 and S100A9 were widely documented in various studies associated with DRYaq, DRYaqlip and SS^21^,^22^,^23^,^25^,^26^. These proteins are identified to play a part in inflammatory processes and Zhou *et al*. demonstrated that higher expressions of these proteins were related with increased signs of dryness^23^. Zhou *et al*. also demonstrated that the increased abundance of ENO1 in the tears is correlated to the DES^23^. ENO1 is an important glycolytic enzyme, hitherto several studies demonstrated their potential roles in several disease progressions, namely, in cancer and autoimmune disorders^58^,^59^,^60^,^61^. The similar increment of ORM1 protein was also documented in DRYaqlip and SS groups^23^,^25^. ORM1, is a heavily glycosylated protein and categorized as a member of the immunocalin family that modulates inflammatory and immune responses^62^. Meanwhile, the similar increment of TF was only documented by Li *et al*. in DRY_SS group^25^. TF is an iron binding transport protein and this characteristic resulted in anti-microbial properties^63^,^64^. PEBP1 was found for the first time significantly increased in abundance in DRYaq and DRYaqlip groups in this study. PEBP1 is an inhibitory modulator of Raf kinase protein and G-protein coupled receptor (GPCR) signalling cascade, as well as an activator of nuclear factor κB (NF-κB)^65^,^66^. Therefore, PEBP1 basically represents a novel effector of signal transduction pathways that modulate apoptosis, motility, therapeutic resistance, genomic integrity and cellular growth^65^. Besides, the disruption of PEBP1 has been reported to be related with a diverse range of diseases, namely, pancreatitis, cancer and Alzheimer’s disease, making it a potential target for disease therapy^65^. Increment in abundance of ALDH3A1 was also documented in several studies of DRY_SS, MDE but has never been verified^24^,^25^. ALDH3A1 is a corneal crystallin protein, highly expressed in epithelial cells and stromal keratocytes^67^. ALDH3A1 plays an active metabolism of toxic aldehydes in the cornea^68^. An essential role for ALDH3A1 in the cornea is suggested by observation that diseased corneas, especially cataract development, are associated with decreased ALDH3A1 catalytic activity^69^,^70^.

The rest of the 66 differentially expressed proteins were not successfully verified, largely due to the low abundance of these proteins in tears and limitations of the AIMS strategy. It is well-recognized that in the sequence of new biomarker identification, the validity of potential candidate biomarker panels must be tested thoroughly in individual samples. However, biomarker validation is a very costly and time-consuming task, primarily due to the complexity of multiplexed assays, as well as technological challenges in identifying and quantifying low abundant proteins^71^,^72^. Hence, in the current study, the AIMS analysis employed was limited to the pooled tear samples to screen for and verify major differentially expressed proteins in DES samples. However, since this is the first study that extensively identified and verified the cluster of differentially expressed proteins in tears, which could be potential biomarker candidate(s) for the different subgroups of DES. The important findings emerging from this this study with the use of pooled samples are envisioned to provide fundamental information and will be a highly useful platform and reference point for future studies utilizing individual samples to dissect ‘personalized’ expression of these proteins. This will be especially useful for analysis of individual tear samples of larger cohorts of DES patients in the clinical settings to demonstrate the discriminative power (e.g. sensitivity and specificity).

In conclusion, this study had unravelled the intricate regulation profiles of specific cluster of proteins responsible for the maintenance of tear film stability in the different DES subgroups. Besides, we have unravelled numerous proteins that participate in metabolic processes, which might provide new insights for modulating various biological processes, especially the inflammatory and immune defence disorders of the ocular surface. The outcomes of the identification of these proteins, when extrapolated to clinical application, can provide invaluable hints on development of specific diagnostic tool for clinical tests and are of great importance for the prognostic usage for improved clinical management of the disease in the future.

## Materials and Methods

### Tear sampling

The study was performed in strict adherence to the guidelines of the 1964 Declaration of Helsinki and all experimental protocols were approved by the “Landesärztekammer Rheinland-Pfalz” ethics committee. All participants were informed of the possible risks, the goal of the study and privacy policy, and an informed consent was signed according to the recommendations of the Declaration of Helsinki for investigation with human subjects. In this study, tear proteins of 80 patients were included and assigned into DRYlip, DRYaq, DRYaqlip and CTRL. Each group comprises 20 subjects equally divided to male (M) and female (F), age between 21 to 79 years old. The classification of patients was carried out according to the guidelines of the Tear Film and Ocular Surface Society (TFOS, Ocular Surface, 2007) at the Department of Ophthalmology at the University Medical Center of the Johannes Gutenberg University, Mainz, following specific inclusion and exclusion criteria of basic secretory test (BST), tear breakup time (TBUT), clinical parameters after Bron and Foulks Score and Lid-parallel Conjunctival Folds (LIPCOF) and extensively asking for the anamnesis and symptoms using Ocular Surface Disease Index (OSDI) questionnaire. Table 3 summarizes the clinical evaluation parameters for the classification DES subgroups and CTRL patients (complete description in Supplementary data 1G). From each individual, tear samples have been taken by a BST, a clinical standard test, using Schirmer’s strip. Schirmer’s strips have been placed into the lower eye lid for a few minutes, after having prepared the eye with mild anesthetic eye drops (Novesine). The eye drops were used in this study to assess the basal tear secretion from the patients as critically as possible. The samples were immediately stored at −80 °C for subsequent analysis. Tear proteins were extracted from the Schirmer’s strips by soaking each strip in 500 μl phosphate-buffered saline (PBS) for 3 hours at 4 °C to elute the tear proteins. Subsequently, the total protein concentrations in the collected tear samples were determined using BCA Protein Assay Kit (Pierce, Rockford, IL) prior to further analysis. Supplementary data 1H summarizes the general description of the study samples and the total protein concentration yielded from DES subgroups and CTRL patients.

### Discovery study: LFQ analysis *via* 1DE & LC-ESI-MS/MS strategy

Label-free quantification of peptides *via* 1DE & LC-ESI-MS/MS strategy was employed to identify the change in protein abundance in the discovery data and to generate a list of the differentially expressed proteins from specific DES subgroups for subsequent verification. The tear samples for each assigned group (N = 20) were pooled equally (2.5 μg per individual) to a total of 50 μg with three replicates. The reason for equal amount of protein collection and pooling from each patient within a group was to normalize the difference between subjects and to reduce individual variation. The pooled tear samples of each DES subgroups were subjected to 1DE (50 μg/well and sliced into 10 bands), trypsin digested and the extracted peptides were purified utilizing methods previously described^35^,^50^. The LC-ESI-LTQ-Orbitrap MS system employed and the mass spectrometric settings utilized for this study has been described in detail elsewhere^35^,^50^. The acquired continuum MS spectra were analysed by MaxQuant computational proteomics platform version 1.4.1.2 and its built-in Andromeda search engine for peptide and protein identification, with LFQ and iBAQ algorithm enabled^38^,^73^,^74^,^75^. The tandem MS spectra were searched against Uniprot Human database (date, 19/March/2014) using settings as described in detail elsewhere^50^. The false discovery rate (FDR) for protein identification was set to 0.01 with ≥6 amino acid residues and “unique plus razor peptides” were selected to be included for LFQ and iBAQ analysis^73^. The output of the generated “proteingroups.txt” data from the MaxQuant analysis was utilized for Pearson correlation, clustering and statistical analysis using Perseus software. Unsupervised hierarchical clustering of the LFQ values was performed based on Euclidean distances on the Z-scored between mean values. For statistical analysis, two-samples t-test-based statistics with P < 0.01 was applied on Log2 transformed LFQ values and the minimum number of values “in at least one group” is 3 to assert proteins regulation as significant for the specific groups^38^,^50^. The iBAQ values, which were calculated by dividing the summed peptide intensities for a given protein with the number of theoretically observable tryptic peptides, act as a degree of protein abundance were converted to percentage and employed to compare between proteins in each group.

### Verification Study: Targeted MS *via* AIMS strategy

The identified candidate biomarkers from the discovery stage were verified employing a targeted form of MS strategy called AIMS^71^,^76^. The main aim of employing the AIMS strategy for verification in the present study is to determine the major differentially expressed proteins to discriminate the specific DES subgroups. The tear samples for each assigned group (N = 20) were pooled equally (0.5 μg per individual) to a total of 10 μg with quadruplicate and subjected to in-solution digestion. The pooled tear samples were digested with sequence grade-modified trypsin [protease: protein ratio of 1:20 (w/w)] for 16 hours at 37 °C with 50 μl of 50 mM NH_4_HCO_3_ in 10% acetonitrile. The digested samples were purified on ZipTip C18 columns and the eluates were concentrated to dryness in the SpeedVac concentrator and dissolved with 10 μl of 0.1% TFA solution prior to targeted MS analysis^35^,^50^. For targeted MS analysis, the LC-ESI-MS/MS system was operated as described in detail previously with several parameters adjusted to target only a specific set of peptides^35^,^50^. The selection of the experimentally identified peptides list that annotated to specific protein was carried out manually from the “msms.txt” file resulting from MaxQuant analysis governed by these criterions, contains no missed cleavages, fully tryptic, no modifications and peptide only assume charge +2. The created peptide inclusion list comprising *m/z* and charge was assigned to the instrument acquisition software before data acquisition. The in-solution digested tear samples consisted of very complex mixtures especially the high abundant proteins. Hence, to yield optimum results, two LC gradients of 60 min (as described elsewhere^35^) and 120 min were utilized. The gradient for 120 min per sample is as follows: 0–5 min: 10% B, 5–95 min: 10–50% B, 95–105 min: 50–90% B, 105–110 min: 90% B, 110–115 min: 90–10% B, 115–120 min: 10% B. The adjusted parameters for inclusion list-dependent acquisition were as follows: the dynamic exclusion segment was disabled, the use of global parent list was enabled and the *m/z* tolerance around targeted precursors was ±10 ppm. The acquired MS spectra were analysed by MaxQuant for peptide identification by searched against Uniprot Human database (date, 19/March/2014) using settings with peptide mass tolerance of ±10 ppm, fragment mass tolerance of ±0.5, peptide charge state of +2 and FDR for peptide identification was set to 0.01. The output data of the generated ‘peptides.txt’ file from MaxQuant were used to calculate the sum absolute intensity of the signature peptides for each proteins, and were transferred to Statistica (v8, StatSoft, Tulsa, OK) for t-test analysis (independent, by groups, P < 0.05).

### Functional annotation and pathways analysis

Proteins determined to be differentially expressed as described based on the data in our LFQ experiments were tabulated in Excel and their gene names were used for functional annotation and pathways analysis. First, DAVID tool (version 6.7) (http://david.abcc.ncifcrf.gov/home.jsp) was used for interpreting the GOBP terms of the differentially-expressed proteins^77^,^78^. The protein list was uploaded into DAVID and searched for enrichment for GOBP term and the results were filtered based on threshold count ≥2 and P values < 0.05. Ingenuity Pathways Analysis software (IPA, Ingenuity QIAGEN Redwood City, CA) (www.qiagen.com/ingenuity) was used for interpreting the GOCC terms, molecule types and PPI networks as well as top diseases and functions associated with the differentially-expressed proteins. Top canonical pathways involving the differentially expressed proteins were presented, along with a *p*-value calculated using Fisher’s exact test. The molecular interactions networks between proteins associated with top diseases and functions were reported. Proteins are displayed with their corresponding gene names and represented as nodes, whereas protein–protein interactions based on direct associations (experimentally observed) between two nodes are represented with an edge (line). Nodes are presented using different shapes to represent the functional protein class and node colour indicates decreased (green) or increased (red) abundance.

## Additional Information

**How to cite this article**: Perumal, N. *et al*. Proteomics analysis of human tears from aqueous-deficient and evaporative dry eye patients. *Sci. Rep.* **6**, 29629; doi: 10.1038/srep29629 (2016).

## Supplementary Material

Supplementary Information

## Figures and Tables

**Table 1:** The list of the significantly altered PPI networks and their related top diseases and functions analysed employing the Ingenuity Pathways Analysis software in DES subgroups compared to CTRL.

Groups	Increment	Decrement	Score	Focus molecules	Top diseases and functions
DRYlip *vs*. CTRL	IGHA1, SCGB1D1, SCGB2A1	ALB, CSTB, HP, IGHG1, KRT1, KRT9, KRT10, ORM2, SERPINA1	30	12	Dermatological diseases and conditions, developmental disorders
	A2M, ENO1, IGLL1/IGLL5, LCN2	AKR1A1, ANXA2, GDI2, HP, HSPB1, SERPINB1, ZG16B	27	11	Digestive system development and function
DRYaq *vs*. CTRL	ALB, ANXA1, ANXA5, CFH, GC, GSN, HP, IGHG1, IGHG2, MDH1, ORM1, ORM2, PFN1, PPIA, S00A8, S100A9, SERPINA1, TF, TTR	ACTB, CLU, HSPA1A/HSPA1B, HSPG2, IGHA1, KRT1, KRT9, S100A9, ZG16B	71	28	Cellular movement, immune cell trafficking, metabolic disease
	A2M, ASS1, CSTB, FABP5, FBP1, GLOD4, PIGR	CTSB, DMBT1, HSPB1, IGHA2, LACRT, PKM, PRR4, SCGB1D1	31	15	Hereditary disorder, neurological disease, organismal injury and abnormalities
	AKR1C1/AKR1C2, ALDH3A1, ANXA3, ENO1, GSTP1, PARK7, PEBP1, PGK1, PRDX1, SFN	ALDH1A1, AZGP1, TCN1	26	13	Small molecule biochemistry, cancer, endocrine system disorders
DRYaqlip *vs*. CTRL	ALB, ANXA1, CFH, IGHG1, IGHG2, ITIH4, ORM1, PARK7, PIGR, RBP4, S100A8, S100A), S100A11, TF, TPI1, YWHAZ	AZGP1, CLU, DMBT1, HSPG2, IGHA1, KRT1, KRT9, KRT10, LTF, LYZ, MDH1, TCN1	73	29	Antimicrobial response, inflammatory response, humoral immune response
	A2M, ALDOA, ANXA2, ANXA5, CSTB, ENO1, GSN, GSTP1, PFN1, PPIA, SERPINB1, SFN, TGM2	ACTB, CTSB, HSPB1, PGK1	36	17	Cellular movement, inflammatory response, neurological disease
	AKR1A1, FABP5, GLOD4, PEBP1, PRDX1	CST4, LACRT, LYZ, PKM, PRR4, SCGB1D1, SCGB2A1, ZG16B	25	13	Carbohydrate metabolism, small molecule biochemistry, cardiovascular disease

**Table 2:** Summary of the significantly differentially expressed proteins in DES subgroups compared to CTRL employing the targeted proteomics strategy.

Gene names	Peptides [AA-sequences]	m/z[Da]	Charge[+]	MZ + [Da]	Score	Two samples t-test; (*Significant P<0.05)					
						DRYlip *vs*. CTRL		DRYaq *vs*. CTRL		DRYaqlip *vs*. CTRL	
						Significant	p value	Significant	p value	Significant	p value
PRR4	FPSVSLQEASSFFQR	865.43	2	1728.847	344	decrement	0.0004	decrement	0.0000	decrement	0.0000
	FPSVSLQEASSFFR	801.40	2	1600.789	340						
ZG16B	YFSTTEDYDHEITGLR	973.94	2	1945.869	127	decrement	0.0016	decrement	0.0000	decrement	0.0000
SCGB2A1	TINSDISIPEYK	690.35	2	1378.698	197	non-significant	0.0588	decrement	0.0008	decrement	0.0000
DMBT1	FGQGSGPIVLDDVR	730.38	2	1458.747	187	non-significant	0.1682	decrement	0.0228	decrement	0.0000
PROL1	FSQAVILSQLFPLESIR	974.55	2	1947.083	200	decrement	0.0081	decrement	0.0001	decrement	0.0000
LACRT	SILLTEQALAK	593.85	2	1185.697	255	non-significant	0.3401	non-significant	0.0521	decrement	0.0436
ALDH3A1	SLEEAIQFINQR	724.38	2	1446.747	135	non-significant	0.1853	non-significant	0.3497	increment	0.0083
ENO1	GNPTVEVDLFTSK	703.86	2	1405.709	155	non-significant	0.2672	increment	0.0256	increment	0.0037
TF	HSTIFENLANK	637.33	2	1272.646	84	non-significant	0.3587	increment	0.0000	increment	0.0000
S100A8	ALNSIIDVYHK	636.85	2	1271.687	142	increment	0.0229	increment	0.0004	increment	0.0000
PEBP1	GNDISSGTVLSDYVGSGPPK	975.48	2	1948.938	98	non-significant	0.7171	increment	0.0007	increment	0.0000
ORM1	YVGGQEHFAHLLILR	876.98	2	1751.947	127	non-significant	0.4548	increment	0.0004	increment	0.0000
S100A9	NIETIINTFHQYSVK	903.97	2	1805.931	342	increment	0.0001	increment	0.0000	increment	0.0000

**Table 3:** Summary of the clinical parameters for the classification of DES subgroups and CTRL patients.

DES group	Clinical parameters			No. Patients	Gender	Age	Protein Conc. (μg/μl)
	BST	TBUT	Bron and Foulks Score				
CTRL	>10 mm/5 min	>10 s	<18	10	Male	51.4 ± 14.78	0.28 ± 0.14
				10	Female	43.8 ± 15.44	0.32 ± 0.10
DRYlip	>10 mm/5 min	<10 s	>18	10	Male	52.9 ± 20.45	0.23 ± 0.09
				10	Female	51.8 ± 18.66	0.28 ± 0.06
DRYaq	<10 mm/5 min	>10 s	<18	10	Male	47.6 ± 15.32	0.18 ± 0.07
				10	Female	49.6 ± 14.74	0.17 ± 0.07
DRYaqlip	<10 mm/5 min	<10 s	>18	10	Male	57.73 ± 19.38	0.19 ± 0.09
				10	Female	58.78 ± 17.42	0.15 ± 0.06
