# Supplementary material for: Proteomics analysis of human tears from aqueous-deficient and evaporative dry eye patients
Source: Sci Rep. 2016 Jul 20;6:29629. doi: 10.1038/srep29629 (PMC4951640; doi:10.1038/srep29629)
Supplement: Supplementary Information [file srep29629-s1.doc]

**SUPPLEMENTARY DATA**

**Proteomics analysis of human tears from aqueous-deficient and evaporative dry eye patients**

Natarajan Perumal^1^, Sebastian Funke^1^, Norbert Pfeiffer^1^ and Franz H. Grus^1*^

**^1^** Department of Ophthalmology, University Medical Center of the Johannes Gutenberg University Mainz, Mainz, Germany.

***Correspondence**:

Prof. Dr. Dr. Franz H. Grus

Head of the Experimental Ophthalmology,

Department of Ophthalmology, University Medical Center of the Johannes Gutenberg University Mainz, Langenbeckstr 1, 55101 Mainz, Germany.

Tel.: +49 6131 17 3328

E-mail address: [grus@eye-research.org](mailto:grus@eye-research.org)

**TABLE OF CONTENTS**

**Supplementary data 1A**

Complete list of the proteins identified from the DES subgroups and CTRL employing the discovery proteomics strategy.

**Supplementary data 1B**

Summary data on the differentially expressed proteins in DES subgroups compared to CTRL employing the discovery proteomics strategy.

**Supplementary data 1C**

Complete list of the over-represented GOBP terms of the differentially expressed proteins analysed employing the DAVID tool in DES subgroups compared to CTRL.

**Supplementary data 1D**

Complete lists of the PPI networks of the significantly differentially expressed tear proteins analysed employing the Ingenuity Pathways Analysis software in DES subgroups compared to CTRL.

**Supplementary data 1E**

Complete data on the differentially expressed proteins in DES subgroups compared to CTRL tears employing AIMS strategy.

**Supplementary data 1F**

Summary of the differentially expressed tear protein profiles in DES subgroups compared to CTRL in the current study in comparison to previous studies in the literature.

**Supplementary data 1G**

Description of the clinical parameters used for the classification of DES subgroups and CTRL patients.

**Supplementary data 1H**

General description and the total protein concentration yielded from the tear samples of DES subgroups and CTRL patients.

Supplementa**r**y data 1A:

Complete list of the proteins identified from the DES subgroups and CTRL employing the discovery proteomics strategy.

**Supplementary data 1A: (continued)**

**Supplementary data 1A: (continued)**

**Supplementary data 1A: (continued)**

**Supplementary data 1B:**

Summary data on the differentially expressed proteins in DES subgroups compared to CTRL employing the discovery proteomics strategy.

**Supplementary data 1B: (continued)**

**Supplementary data 1C:**

Complete list of the over-represented GOBP terms of the differentially expressed proteins analysed employing the DAVID tool in DES subgroups compared to CTRL.

**Supplementary data 1C: (continued)**

**Supplementary data 1C: (continued)**

**Supplementary data 1D:**

Complete lists of the PPI networks of the significantly differentially expressed tear proteins analysed employing the Ingenuity Pathways Analysis software in DES subgroups compared to CTRL.

**Supplementary data 1D: (continued)**

**Supplementary data 1D: (continued)**

**Supplementary data 1D: (continued)**

**Supplementary data 1E:**

Complete data on the differentially expressed proteins in DES subgroups compared to CTRL tears employing AIMS strategy.

**Supplementary data 1F:**

**Summary of the differentially expressed tear protein profiles in DES subgroups compared to CTRL in the current study in comparison to previous studies in the literature.**

| **Gene names** | **Expression profiles in this study** | | | **Expression profiles from literatures** | |
| --- | --- | --- | --- | --- | --- |
|  | **DRYlip**  **/CTRL** | **DRYaq**  **/CTRL** | **DRYaqlip**  **/CTRL** | **Profiles** | **DES [Citations]** |
| ALB | DOWN | UP | UP | UP | DRY_CL [^1^](#_ENREF_1) |
| AZGP1 | n.s | DOWN | DOWN | DOWN | DRYaq & DRYlip [^2^](#_ENREF_2), DRY_MDE & DRY_MSDE [^3^](#_ENREF_3) |
| CST4 | n.s | n.s | DOWN | DOWN | DRYlip [^2^](#_ENREF_2), DRY_SS [^4^](#_ENREF_4), DRY_MDE & DRY_MSDE [^3^](#_ENREF_3) |
| ENO1 | UP | UP | UP | UP | DRYaqlip [^5^](#_ENREF_5) |
| GSTP1 | n.s | UP | UP | UP | DRYaq & DRYlip [^2^](#_ENREF_2) |
| IGHA1 | UP | DOWN | DOWN | DOWN | DRY_SS [^4^](#_ENREF_4), DRY_MSDE [^3^](#_ENREF_3) |
| LACRT | n.s | DOWN | DOWN | DOWN | DRY_CL [^1^](#_ENREF_1), DRYaq, DRY_pSS, DRY_SJS & DRY_RA [^6^](#_ENREF_6), DRY_SS [^4^](#_ENREF_4), DRY_MDE & DRY_MSDE [^3^](#_ENREF_3) |
| LTF | n.s | n.s | DOWN | DOWN | DRYaq & SS [^7^](#_ENREF_7); DRYaqlip [^5^](#_ENREF_5); DRYaq, DRY_pSS, DRY_SJS & DRY_RA [^6^](#_ENREF_6); DRYaq [^2^](#_ENREF_2), DRY_SS [^4^](#_ENREF_4), DRY_MDE & DRY_MSDE [^3^](#_ENREF_3) |
| LYZ | n.s | n.s | DOWN | DOWN | DRYaqlip [^8^](#_ENREF_8), DRY_SS [^4^](#_ENREF_4), DRY_MDE & DRY_MSDE [^3^](#_ENREF_3) |
| PIGR | n.s | UP | UP | DOWN | DRY_SS [^4^](#_ENREF_4), DRY_MDE & DRY_MSDE [^3^](#_ENREF_3) |
| PRR4 | n.s | DOWN | DOWN | DOWN | DRYaq [^9^](#_ENREF_9); DRY_CL [^1^](#_ENREF_1); DRY_pSS & DRY_SJS [^10^](#_ENREF_10); DRYaq, DRY_pSS, DRY_SJS & DRY_RA [^6^](#_ENREF_6), DRYaq & DRYaqlip [^11^](#_ENREF_11), DRY_SS [^4^](#_ENREF_4), DRY_MDE & DRY_MSDE [^3^](#_ENREF_3) |
| S100A8 | n.s | UP | UP | UP | DRYaq [^9^](#_ENREF_9), DRYaqlip [^5^](#_ENREF_5), DRYaq & DRYaqlip [^11^](#_ENREF_11), DRYaq & DRYlip [^2^](#_ENREF_2), DRY_SS [^4^](#_ENREF_4) |
| S100A9 | n.s | UP | UP | UP | DRYaqlip [^5^](#_ENREF_5), DRYaq & DRYlip [^2^](#_ENREF_2), DRY_SS [^4^](#_ENREF_4) |
| SCGB1D1 | UP | DOWN | DOWN | DOWN  UP | DRY_CL [^1^](#_ENREF_1),  DRYaq & DRYaqlip [^11^](#_ENREF_11), DRY_MDE & DRY_MSDE [^3^](#_ENREF_3) |
| SCGB2A1 | UP | n.s | DOWN | DOWN  DOWN  UP | DRYaq [^2^](#_ENREF_2)  DRY_MDE & DRY_MSDE [^3^](#_ENREF_3)  DRYaq & DRYaqlip [^11^](#_ENREF_11), |
| ACTB | n.s | DOWN | DOWN | DOWN  UP | DRYaq & DRYlip [^2^](#_ENREF_2)  DRY_SS [^4^](#_ENREF_4) |
| ALDH3A1 | n.s | UP | UP | UP | DRY_SS [^4^](#_ENREF_4), DRY_MDE [^3^](#_ENREF_3) |
| ANXA1 | n.s | UP | UP | UP | DRYaq & DRYlip [^2^](#_ENREF_2), DRY_SS [^4^](#_ENREF_4) |
| ANXA2 | DOWN | n.s | UP | UP | DRY_SS [^4^](#_ENREF_4) |
| ANXA5 | n.s | UP | UP | DOWN | DRY_SS [^4^](#_ENREF_4) |
| C3 | n.s | UP | UP | UP | DRY_SS [^4^](#_ENREF_4), DRY_MDE [^3^](#_ENREF_3) |
| CLU | n.s | DOWN | DOWN | UP | DRY_SS [^4^](#_ENREF_4) |
| CSTB | DOWN | UP | UP | DOWN | DRY_SS [^4^](#_ENREF_4) |
| GC | n.s | UP | n.s | UP | DRY_MSDE [^3^](#_ENREF_3) |
| HP | DOWN | UP | n.s | UP | DRY_MDE [^3^](#_ENREF_3) |
| HSPA1A | n.s | DOWN | n.s | UP | DRY_SS [^4^](#_ENREF_4) |

**Supplementary data 1F:**  (continued)

| **Gene names** | **Expression profiles in this study** | | | **Expression profiles from literatures** | |
| --- | --- | --- | --- | --- | --- |
|  | **DRYlip**  **/CTRL** | **DRYaq**  **/CTRL** | **DRYaqlip**  **/CTRL** | **Profiles** | **DES [Citations]** |
| IGHA2 | n.s | DOWN | DOWN | DOWN | DRY_MSDE [^3^](#_ENREF_3) |
| IGJ | n.s | n.s | DOWN | DOWN | DRYaq, DRY_pSS, DRY_SJS & DRY_RA [^6^](#_ENREF_6), DRY_MDE & DRY_MSDE [^3^](#_ENREF_3) |
| KRT1 | DOWN | DOWN | DOWN | UP | DRY_SS [^4^](#_ENREF_4) |
| KRT10 | DOWN | n.s | DOWN | UP | DRY_SS [^4^](#_ENREF_4) |
| ORM1 | n.s | UP | UP | UP | DRYaqlip [^5^](#_ENREF_5), DRY_SS [^4^](#_ENREF_4) |
| PRDX1 | n.s | UP | UP | UP | DRY_MSDE [^3^](#_ENREF_3) |
| PROL1 | n.s | DOWN | DOWN | DOWN | DRY_MSDE [^3^](#_ENREF_3) |
| SERPINA1 | DOWN | UP | n.s | UP | DRYaq [^9^](#_ENREF_9), DRYaq & DRYaqlip [^11^](#_ENREF_11) |
| TCN1 | n.s | DOWN | DOWN | DOWN | DRY_MDE [^3^](#_ENREF_3) |
| TF | n.s | UP | UP | UP | DRY_SS [^4^](#_ENREF_4) |
| ZG16B | DOWN | DOWN | DOWN | DOWN | DRY_MSDE [^3^](#_ENREF_3) |
| IGHG1 | DOWN | UP | UP | New findings | |
| IGLC3 | n.s | n.s | UP | New findings | |
| IGLL5 | UP | n.s | n.s | New findings | |
| A2M | UP | UP | UP | New findings | |
| AKR1A1 | DOWN | n.s | UP | New findings | |
| AKR1C1 | n.s | UP | UP | New findings | |
| ALDH1A1 | n.s | DOWN | n.s | New findings | |
| ALDOA | n.s | n.s | UP | New findings | |
| ANXA3 | n.s | UP | n.s | New findings | |
| ASS1 | n.s | UP | n.s | New findings | |
| CFH | n.s | UP | UP | New findings | |
| CTSB | n.s | DOWN | DOWN | New findings | |
| DMBT1 | n.s | DOWN | DOWN | New findings | |
| FABP5 | n.s | UP | UP | New findings | |
| FBP1 | n.s | UP | UP | New findings | |
| GDI2 | DOWN | n.s | n.s | New findings | |
| GLOD4 | n.s | UP | UP | New findings | |

**Supplementary data 1F:**  (continued)

| **Gene names** | **Expression profiles in this study** | | | **Expression profiles from literatures** |
| --- | --- | --- | --- | --- |
|  | **DRYlip**  **/CTRL** | **DRYaq**  **/CTRL** | **DRYaqlip**  **/CTRL** | **DES [Citations]** |
| GSN | n.s | UP | UP | New findings |
| HSPB1 | DOWN | DOWN | DOWN | New findings |
| HSPG2 | n.s | DOWN | DOWN | New findings |
| IGHG2 | n.s | UP | UP | New findings |
| ITIH4 | n.s | n.s | UP | New findings |
| KRT9 | DOWN | DOWN | DOWN | New findings |
| LCN2 | UP | n.s | n.s | New findings |
| MDH1 | n.s | UP | DOWN | New findings |
| ORM2 | DOWN | UP | n.s | New findings |
| PARK7 | n.s | UP | UP | New findings |
| PEBP1 | n.s | UP | UP | New findings |
| PFN1 | n.s | UP | UP | New findings |
| PGK1 | n.s | UP | DOWN | New findings |
| PKM | n.s | DOWN | DOWN | New findings |
| PPIA | n.s | UP | UP | New findings |
| RBP4 | n.s | n.s | UP | New findings |
| S100A11 | n.s | DOWN | UP | New findings |
| SERPINB1 | DOWN | n.s | UP | New findings |
| SFN | n.s | UP | UP | New findings |
| TGM2 | n.s | n.s | UP | New findings |
| TPI1 | n.s | n.s | UP | New findings |
| TTR | n.s | UP | n.s | New findings |
| UBA1 | n.s | n.s | UP | New findings |
| UBB | n.s | n.s | UP | New findings |
| YWHAZ | n.s | n.s | UP | New findings |

Note: The abbreviations used in this table are as follows; Sjögren syndrome (SS), contact lens-related dry eye (DRY_CL), dry eye associated with Sjogren’s syndrome (DRY_SS), dry eye associated with Stevens-Johnson syndrome (DRY_SJS), dry eye associated with rheumatoid arthritis (DRY_RA), mildly symptomatic with aqueous deficiency (DRY_MDE) and symptomatic aqueous deficiency (DRY_MSDE), non-significant (n.s).

**REFERENCES**

1 Nichols, J. J. & Green-Church, K. B. Mass spectrometry-based proteomic analyses in contact lens-related dry eye. *Cornea* **28**, 1109-1117, doi:10.1097/ICO.0b013e3181a2ad81 (2009).

2 Soria, J. *et al.* Tear proteome and protein network analyses reveal a novel pentamarker panel for tear film characterization in dry eye and meibomian gland dysfunction. *Journal of proteomics* **78**, 94-112, doi:10.1016/j.jprot.2012.11.017 (2013).

3 Srinivasan, S., Thangavelu, M., Zhang, L., Green, K. B. & Nichols, K. K. iTRAQ quantitative proteomics in the analysis of tears in dry eye patients. *Investigative ophthalmology & visual science* **53**, 5052-5059 (2012).

4 Li, B. *et al.* Tear proteomic analysis of Sjögren syndrome patients with dry eye syndrome by two-dimensional-nano-liquid chromatography coupled with tandem mass spectrometry. *Scientific reports* **4** (2014).

5 Zhou, L. *et al.* Identification of tear fluid biomarkers in dry eye syndrome using iTRAQ quantitative proteomics. *Journal of proteome research* **8**, 4889-4905, doi:10.1021/pr900686s (2009).

6 Aluru, S. V. *et al.* Lacrimal proline rich 4 (LPRR4) protein in the tear fluid is a potential biomarker of dry eye syndrome. *PloS one* **7**, e51979, doi:10.1371/journal.pone.0051979 (2012).

7 Ohashi, Y. *et al.* Abnormal protein profiles in tears with dry eye syndrome. *American journal of ophthalmology* **136**, 291-299 (2003).

8 Zhou, L. *et al.* Elevation of human alpha-defensins and S100 calcium-binding proteins A8 and A9 in tear fluid of patients with pterygium. *Invest Ophthalmol Vis Sci* **50**, 2077-2086, doi:10.1167/iovs.08-2604 (2009).

9 Grus, F. H. *et al.* SELDI-TOF-MS ProteinChip array profiling of tears from patients with dry eye. *Invest Ophthalmol Vis Sci* **46**, 863-876, doi:10.1167/iovs.04-0448 (2005).

10 Saijyothi, A. V. *et al.* Two dimensional electrophoretic analysis of human tears: collection method in dry eye syndrome. *Electrophoresis* **31**, 3420-3427, doi:10.1002/elps.201000271 (2010).

11 Boehm, N. *et al.* Alterations in the Tear Proteome of Dry Eye Patients—A Matter of the Clinical PhenotypeTear Proteome of Dry Eye Patients. *Investigative ophthalmology & visual science* **54**, 2385-2392 (2013).

**Supplementary data 1G:**

Description of the clinical parameters used for the classification of DES subgroups and CTRL patients.

**1.        DRYaq:**

Patients with a BST ≤ 10 mm/ 5 min and no pathologic TBUT of > 10 s were classified as aqueous deficient patients, due to the fact that a reduced production/secretion of tear fluid will predominantly result in a lower BST value [1, 2]. With the purpose to get a representative insight into the tear proteome of patients suffering from different stages of aqueous deficient dry-eye, we chose a BST threshold of ≤ 10 mm/ 5 min, in accordance with previous studies of our group and others **[3-5]**.

**2.        DRYlip:**

Patients with a TBUT ≤ 10 s and a BST value of > 10 mm/ 5 min were classified as evaporative dry eye. A dysfunctional, thinned lipid layer goes along with a pathological/ decreased TBUT [6], which is due to the lower tear film stability [7]. However, the TBUT is not necessarily correlated with a decreased BST value [8]. Thus, in the present study patients with a BST value > 10 mm/ 5 min and a TBUT ≤ 10 s were specifically classified as lipid deficient dry-eye (DRYlip) subjects, as they reveal a normal aqueous state but a pathological tear film stability. Further, the occurrence of a lipid layer deficiency was assured by inspecting meibomian glands [9]. Patients with a score ≥ 18 were classified as pathologic, respectively lipid deficient. Foulks and Bron suggested a threshold of > 10 [9]. Due to the higher specificity, we set a threshold of ≥ 18.

**3.        DRYaqlip:**

Patients with a BST value ≤10 mm/ 5 min and a TBUT ≤10 were classified as patients who suffer from a combined pathogenesis, means they are aqueous deficient as well as lipid deficient. It is generally accepted that symptoms of advanced stages of aqueous deficiency and lipid deficiency are overlapping and influencing each other. As suggested by Versura et al, a thin lipid layer results in an unstable tear film and an aqueous tear-deficient state [5]. Thus, we classified patients with a pathologic BST, TBUT and optionally a score ≥18 as aqueous and lipid deficient.

**4.        Excluded patients from this study were as follows:**

Patients suffering from Sjögren's syndrome, diabetes mellitus, allergy to local anesthetics and contact lens wearers. Patients who had undergone ophthalmic surgery during the last 6 months. Further, the use of any systemic drug suspected to have an influence on the tear production or systemic inflammatory processes led to exclusion of subjects (e.g., beta-blockers, selective serotonin reuptake inhibitors, oral contraceptives, postmenopausal estrogen therapy, local sympathomimetic drugs, nonsteroidal antirheumatic drugs [NSAR analgesics, such as ibuprofen, diclofenac, Voltaren, aspirin, Novalgin, Arcoxia], steroidal antirheumatic drugs, antihistamines).

**REFERENCES**

[1] Gipson IK: Research in dry eye: report of the Research Subcommittee of the International Dry Eye WorkShop (2007). The Ocular Surface 2007, 5(2):179-193.

[2] Lemp MA, Foulks GN: The definition and classification of dry eye disease. The Ocular Surface 2007, 5(2):75-92.

[3] Grus FH, Augustin AJ: Analysis of tear protein patterns by a neural network as a diagnostical tool for the detection of dry eyes. From Genome to Proteome: Advances in the Practice and Application of Proteomics:295-300.

[4] Grus FHA, A. J: High performance liquid chromatography analysis of tear protein patterns in diabetic and non-diabetic dry-eye patients European Journal of Ophthalmology 2001, 11(1).

[5] Versura P, Nanni P, Bavelloni A, Blalock WL, Piazzi M, Roda A, Campos EC: Tear proteomics in evaporative dry eye disease. Eye (London, England) 2010, 24(8):1396-1402.

[6] Craig JP, Tomlinson A: Importance of the lipid layer in human tear film stability and evaporation. Optometry & Vision Science 1997, 74(1):8-13.

[7] Maïssa C, Guillon M: Tear film dynamics and lipid layer characteristics—Effect of age and gender. Contact Lens and Anterior Eye 2010, 33(4):176-182.

[8] Tong L, Chaurasia SS, Mehta JS, Beuerman RW: Screening for meibomian gland disease: its relation to dry eye subtypes and symptoms in a tertiary referral clinic in singapore. Investigative ophthalmology & visual science 2010, 51(7):3449-3454.

[9] Foulks GN, Bron AJ: Meibomian gland dysfunction: a clinical scheme for description, diagnosis, classification, and grading. The ocular surface 2003, 1(3):107-126.

**Supplementary data 1H:**

General description and the total protein concentration yielded from the tear samples of DES subgroups and CTRL patients.

| **Group** | **Patients ID** | **Gender** | **Age** | **Protein Conc. (μg/μl)** |
| --- | --- | --- | --- | --- |
| CTRL | A1 | M | 26 | 0.39 |
| CTRL | A2 | M | 73 | 0.13 |
| CTRL | A3 | M | 59 | 0.34 |
| CTRL | A4 | M | 45 | 0.60 |
| CTRL | A5 | M | 40 | 0.27 |
| CTRL | A6 | M | 35 | 0.22 |
| CTRL | A7 | M | 61 | 0.34 |
| CTRL | A8 | M | 52 | 0.23 |
| CTRL | A9 | M | 56 | 0.18 |
| CTRL | A10 | M | 67 | 0.14 |
| CTRL | A11 | F | 65 | 0.18 |
| CTRL | A12 | F | 52 | 0.23 |
| CTRL | A13 | F | 49 | 0.31 |
| CTRL | A14 | F | 30 | 0.36 |
| CTRL | A15 | F | 24 | 0.34 |
| CTRL | A16 | F | 67 | 0.32 |
| CTRL | A17 | F | 23 | 0.49 |
| CTRL | A18 | F | 41 | 0.18 |
| CTRL | A19 | F | 39 | 0.41 |
| CTRL | A20 | F | 48 | 0.41 |
|  |  | **Average** | **47.60** | **0.30** |
|  |  | **S.D** | **15.22** | **0.12** |
|  |  |  |  |  |
| **Group** | **Patients ID** | **Gender** | **Age** | **Protein Conc. (μg/μl)** |
| DRYaq | B1 | M | 31 | 0.23 |
| DRYaq | B2 | M | 28 | 0.12 |
| DRYaq | B3 | M | 43 | 0.24 |
| DRYaq | B4 | M | 67 | 0.17 |
| DRYaq | B5 | M | 55 | 0.32 |
| DRYaq | B6 | M | 70 | 0.17 |
| DRYaq | B7 | M | 60 | 0.11 |
| DRYaq | B8 | M | 37 | 0.09 |
| DRYaq | B9 | M | 52 | 0.21 |
| DRYaq | B10 | M | 33 | 0.14 |
| DRYaq | B11 | F | 59 | 0.25 |
| DRYaq | B12 | F | 65 | 0.09 |
| DRYaq | B13 | F | 42 | 0.13 |
| DRYaq | B14 | F | 54 | 0.11 |
| DRYaq | B15 | F | 23 | 0.10 |
| DRYaq | B16 | F | 44 | 0.22 |
| DRYaq | B17 | F | 34 | 0.17 |
| DRYaq | B18 | F | 73 | 0.18 |
| DRYaq | B19 | F | 54 | 0.17 |
| DRYaq | B20 | F | 48 | 0.29 |
|  |  | **Average** | **48.60** | **0.18** |
|  |  | **S.D** | **14.67** | **0.07** |

**Supplementary data 1H**: (continued)

| **Group** | **Patients ID** | **Gender** | **Age** | **Protein Conc. (μg/μl)** |
| --- | --- | --- | --- | --- |
| DRYlip | C1 | M | 27 | 0.26 |
| DRYlip | C2 | M | 76 | 0.14 |
| DRYlip | C3 | M | 44 | 0.40 |
| DRYlip | C4 | M | 38 | 0.18 |
| DRYlip | C5 | M | 74 | 0.17 |
| DRYlip | C6 | M | 21 | 0.22 |
| DRYlip | C7 | M | 67 | 0.19 |
| DRYlip | C8 | M | 44 | 0.21 |
| DRYlip | C9 | M | 69 | 0.13 |
| DRYlip | C10 | M | 69 | 0.35 |
| DRYlip | C11 | F | 34 | 0.39 |
| DRYlip | C12 | F | 52 | 0.22 |
| DRYlip | C13 | F | 79 | 0.25 |
| DRYlip | C14 | F | 63 | 0.18 |
| DRYlip | C15 | F | 69 | 0.31 |
| DRYlip | C16 | F | 49 | 0.26 |
| DRYlip | C17 | F | 45 | 0.31 |
| DRYlip | C18 | F | 25 | 0.30 |
| DRYlip | C19 | F | 30 | 0.34 |
| DRYlip | C20 | F | 72 | 0.23 |
|  |  | **Average** | **52.35** | **0.25** |
|  |  | **S.D** | **19.06** | **0.08** |
|  |  |  |  |  |
| **Group** | **Patients ID** | **Gender** | **Age** | **Protein Conc. (μg/μl)** |
| DRYaqlip | D1 | M | 70 | 0.13 |
| DRYaqlip | D2 | M | 63 | 0.16 |
| DRYaqlip | D3 | M | 79 | 0.15 |
| DRYaqlip | D4 | M | 24 | 0.20 |
| DRYaqlip | D5 | M | 75 | 0.07 |
| DRYaqlip | D6 | M | 75 | 0.11 |
| DRYaqlip | D7 | M | 67 | 0.08 |
| DRYaqlip | D8 | M | 69 | 0.12 |
| DRYaqlip | D9 | M | 65 | 0.18 |
| DRYaqlip | D10 | M | 65 | 0.12 |
| DRYaqlip | D11 | F | 70 | 0.17 |
| DRYaqlip | D12 | F | 23 | 0.11 |
| DRYaqlip | D13 | F | 46 | 0.15 |
| DRYaqlip | D14 | F | 70 | 0.12 |
| DRYaqlip | D15 | F | 51 | 0.04 |
| DRYaqlip | D16 | F | 47 | 0.14 |
| DRYaqlip | D17 | F | 64 | 0.25 |
| DRYaqlip | D18 | F | 66 | 0.18 |
| DRYaqlip | D19 | F | 52 | 0.20 |
| DRYaqlip | D20 | F | 75 | 0.26 |
|  |  | **Average** | **60.80** | **0.15** |
|  |  | **S.D** | **15.79** | **0.06** |
